# The MAX IV imaging concept

**DOI:** 10.1186/s40679-016-0029-7

**Published:** 2016-12-01

**Authors:** Zdeněk Matěj, Rajmund Mokso, Krister Larsson, Vincent Hardion, Darren Spruce

**Affiliations:** MAX IV Laboratory, Lund University, Fotongatan 2, Lund, Sweden

**Keywords:** X-ray imaging, Computed tomography, Data acquisition, Image reconstruction

## Abstract

The MAX IV Laboratory is currently the synchrotron X-ray source with the beam of highest brilliance. Four imaging beamlines are in construction or in the project phase. Their common characteristic will be the high acquisition rates of phase-enhanced images. This high data flow will be managed at the local computing cluster jointly with the Swedish National Computing Infrastructure. A common image reconstruction and analysis platform is being designed to offer reliable quantification of the multidimensional images acquired at all the imaging beamlines at MAX IV.

## Background

The MAX IV Laboratory is a synchrotron radiation facility, administered as part of Lund University and was inaugurated on June 21, 2016 with an initial portfolio of 14 experimental instrument stations, called beamlines. These cover mainly spectroscopy and diffraction with full-field imaging capabilities to be added in the following few years. The two diffraction limited storage rings of MAX IV operating at electron energies of 1.5 and 3 GeV [1] have 30 straight sections to be allocated to beamlines. The first beamlines to produce imaging data will be the NanoMAX, SoftiMAX and DanMAX, followed later by BioMedMAX.

The computing infrastructure of MAX IV is being designed to support the operation of the imaging beamlines with continuously increasing complexity, peak data rates and data processing. Imaging beamlines have high demands on the data management infrastructure for both fast “on-site” and detailed “offline” data analysis. Considering developments in other hard X-ray techniques (current trends in macromolecular and serial crystallography, sub-second time resolved spectroscopy, small angle scattering and diffraction), the amount of raw data produced by hard X-ray detectors in MAX IV is expected to be of a similar scale. All the beamlines are supported to adopt the best practice in the computation techniques available in the relevant fields, utilizing common solutions if possible. MAX IV has adopted the well-established hierarchical data format (HDF5) [2] using the NeXus standard [3] where possible. There are various alternative solutions for data representation such as the Argon National Laboratory DataExchange [4] or CXI format [5] for coherent imaging data. It has also become evident that accessing data with low latency through smart-data streams is becoming an important requisite for an effective use of the next-generation light sources. Developments at various synchrotron facilities were reacting to this need in the past years, an example being the new GigaFRoST camera system [6] at the Paul Scherrer Institut or the current developments in data acquisition and analysis systems for X-ray free electron lasers (e.g., [7]).

The MAX IV Laboratory is hosted by the Lund University. Therefore, the computing resources of the Lund University Center for Scientific computing (Lunarc) [8] are accessible via the Swedish National Infrastructure for Computing (SNIC) [9]. Similar connections are foreseen to Danish imaging infrastructures located in the Copenhagen region nearby. This should intensify collaboration on computational methods and tools.

## The MAX IV imaging beamlines

The main focus area of NanoMAX and SoftiMAX will be coherent diffraction imaging and scanning microscopy in hard and soft X-ray regions, respectively.

NanoMAX is a hard X-ray nanoprobe beamline with two instruments under development. First is the scanning X-ray microscopy and diffraction station using a pair of Kirkpatrick–Baez mirrors focusing the beam down to 50–200 nm. With 10^12^ photons/s on sample at 10 keV this instrument will fully utilize the highly coherent flux of the MAX IV source. The second instrument is based on Fresnel zone plate optics focusing down to 10 nm. Both instruments will be able to deliver 3D datasets with the main characteristic of being composed of rather few angular projections. The scanning methods will be in general slower than full-field imaging.

SoftiMAX is designed to be a two-branch soft X-ray (275–2500 eV) beamline with the first branch designed for scanning transmission X-ray microscopy (STXM) including ptychography. The beamline will utilize very high flux at shorter wavelengths from the 3-GeV ring with photon beam focused to 10–100 nm. The second branch will be a modular coherent X-ray imaging (CXI) station.

The DanMAX beamline will combine diffraction and full-field micrometer scale imaging mainly oriented to in situ experiments with hard X-rays (10–40 keV). Fast CMOS detectors are expected to deliver several GB of data per second. The natural workflow will include near-field (Fresnel) phase reconstruction routines coupled to tomographic reconstruction.

BioMedMAX will be the first MAX IV beamline fully dedicated to full-field imaging in the Fresnel diffraction regime with hard X-rays (12–40 keV). With emphasis on studying processes in biological systems at the micrometer scale, advanced acquisition triggering will be of high importance. Similarly to DanMAX, this beamline is expected to deliver typically well-sampled Fourier space in the tomographic settings with rather high noise content as a consequence of radiation dose optimization and short exposure times.

## Controlling the image acquisition

### Sardana control system

Sardana [10] is a software suite for Supervision, Control and Data Acquisition (SCADA) in scientific installations. It aims to reduce cost and time of design, development and support of the control and data acquisition (DAQ) systems. Sardana development was started at the ALBA synchrotron (Spain) and designed using a large experience from ESRF beamlines. Today, it is used and supported by a larger community which includes several other laboratories (ALBA, DESY, MAX IV, Solaris). Sardana was evaluated at the Max-lab facility for a potential use at the next MAX IV Laboratory. After the completion of the MAX IV Laboratory, it became the preferred platform even though its immaturity in terms of well-developed user friendliness was apparent. It was foreseen that the community would improve this as its use became more widespread. The main desirable characteristics of Sardana are as follows:

- The same programming language (python) is used to implement the framework, resulting in a more accessible application and lower complexity. This was thought to have a positive impact for the user autonomy. Python is very widespread both in the control system and scientific software domains which gives more accessibility to these communities.
- Sardana is fully integrated with Tango [11] and therefore complementing the Tango standard which was already chosen for MAX IV.
- Taurus [12] provided an easy way to build simple or complex graphical panels with easy access methods for displaying data acquisition results and control of the instrumentation, making it versatile for all accelerator and beamline graphical interfaces.

### Control with virtual machines

Maintenance of computer hardware in a research facility can be a difficult task because of the widespread distribution of components, sometimes difficult to access. This can lead to issues related to maintaining good reliability and reducing down time caused by computer component failures. In a distributed control system, a controller is present near every sensor or actuator and traditionally requires some computer locally connected via an interface. In the MAX IV control system architecture, the hardware controller manages the hard real-time operation. In combination, all soft real-time operations are delegated to the higher level control computing. Nowadays, more and more connections from devices are made using Ethernet, permitting easier management of the compute resources by relocating the control computing hardware elements into a virtual machine cluster. In this way, only the hardware controller remains distributed, which reduces the distribution of complex computer systems, therefore improving reliability. Other advantages include centralized management with features, such as automatic fail-over of control virtual machines, remote software configuration of machine CPUs, and storage and easy online upgrade of the overall capacity when needed, e.g., adding more dedicated CPU when the software needs more resources.

### Integrating new elements

The Sardana/Tango SCADA is designed for straightforward integration of new components. Area X-ray detectors are the most complex elements of the imaging DAQ as they are expected to produce the crucial data. Their integration into the acquisition process has three layers.

**Fig. 1:**
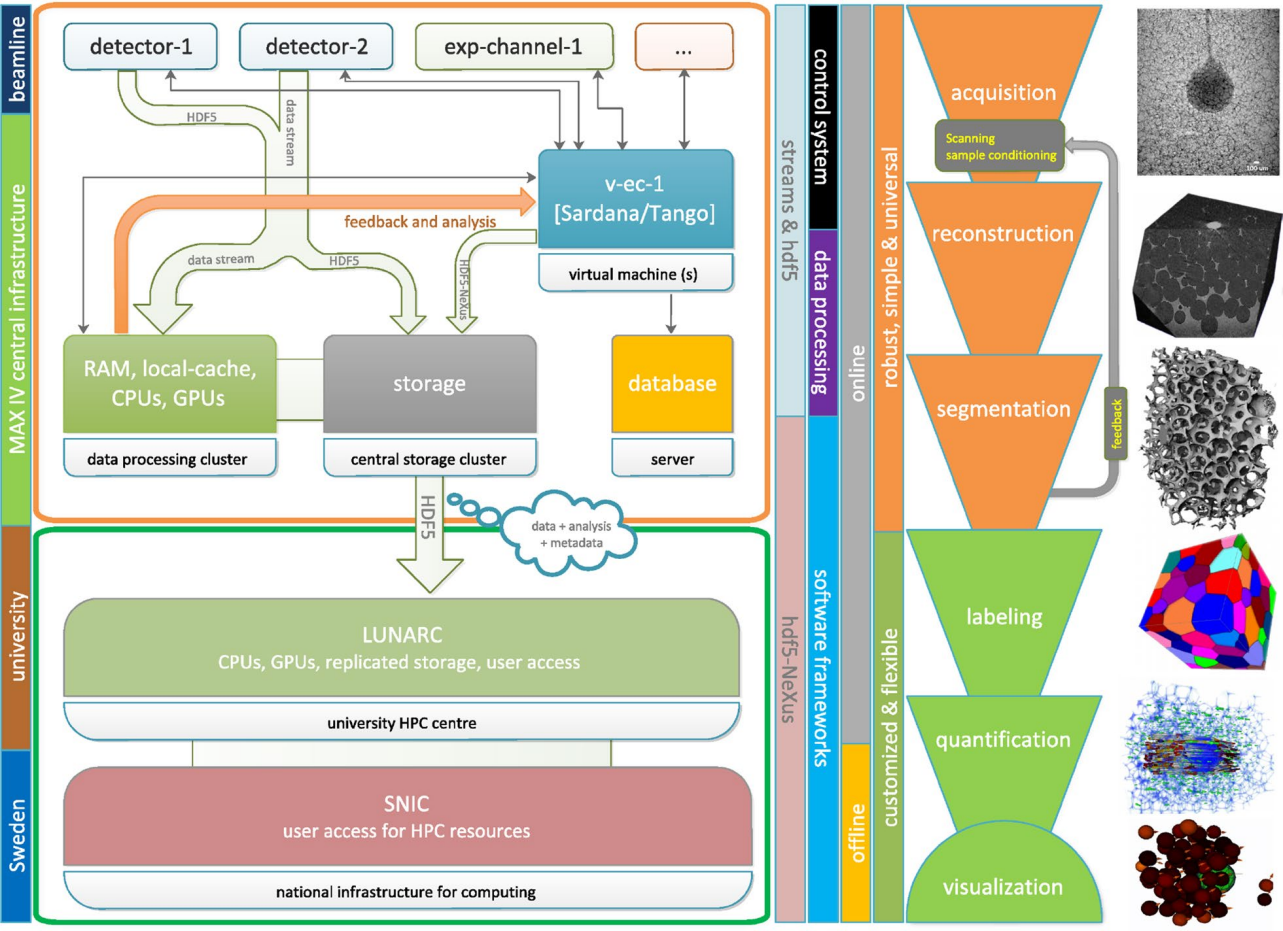
A schematic representation of the imaging data flow concept at MAX IV (on the *left*). The corresponding sequence of imaging data processing methods is depicted on the *right side* of the diagram

1. In the lowest control layer, the specific device control protocol is adapted to Tango by an intermediate LIMA (Library for Image Analysis) system (initially an ESRF development) [13], providing a generic Tango interface for the class of “Detector” (2D or 1D). Other non-generic Tango interfaces can be developed when need to access a specific set of features.
2. Sardana is the second layer in the acquisition work flow which orchestrates acquisitions and data recording. Hardware devices to control are divided into categories (actuators, sensors, counters, etc.) associated with a generic programmable interface. It ensures that data are acquired without including too much specific control logic in the code that executes the sequence. Equipment incompatible with the conventional detector interface defined by LIMA is transparently handled by specific extensions. The role of Sardana is also to make sure data are formatted and stored in a safe space.
3. The last layer of the acquisition process provides raw data visualization and processing. The visualization channel uses Sardana and LIMA interfaces to which any suitable plotting software can be connected. Besides this basic data visualization concept, MAX IV aims to provide a platform for handling (imaging) data with advanced methods and algorithms already during the experiment to maximize effective use of the experimental time. In its most cutting edge variants, it is on-the-fly data handling with feedback to experiment in the form of already interpreted data [6, 7, 14]. The platform for handling imaging data will be running at the central computing infrastructure (see Fig. 1). From the control point of view, the first task is pushing data down in the scheme from the beamlines in Fig. 1. The protocols depend on capabilities of detector systems. Processing HDF5 data with low latencies is always possible today but huge improvement can be achieved using the LIMA interface also for data transfer or streaming protocols supported directly in the detectors. We refer here to current developments as GigaFRoST [6] or trends in high data rate macromolecular crystallography [15]. Another control task is providing a feedback from fast data analysis done at the computing infrastructure to the beamline control system (as indicated in Fig. 1). Finally, the important role of Sardana is also managing the master experimental HDF5/NeXus data file and making links to HDF5 data provided by detectors or processed from the streams.

## Data flow handling

The overall data flow from the imaging experimental stations is schematically depicted in Fig. 1. It regards the needs of diverse imaging methods, as presented in the right part of the picture. Opposite, the available local and national infrastructure is indicated in the left part of Fig. 1. Data are pushed from detectors in the top part of the schema down to the central MAX IV computing and storage infrastructure and further to high-performance computing networks in the bottom.

With the high brightness of the new light source, it is expected to use high frame rate capabilities of modern detectors producing steady data flows in the range of 1–10 Gbit/s and even more in case of tomography. MAX IV beamlines are installed with 2 × 10 Gbit/s connection to the central infrastructure. Moreover this can be extended, as e.g., in the case of a crystallography beamline—at the moment to 40 Gbit/s for a 16M Eiger detector.

The standard data format at MAX IV is HDF5 [2] adding the NeXus convention [3] as an option. Compression will be used wherever possible to reduce the volume. Data from detectors, other experimental channels and results of the preliminary analysis done during the experiment will be complemented with metadata, including information about, e.g., the proposal team. MAX IV will keep these data on site for a maximum of 3 months for transfer to the users’ home institute or remote computing/storage center. However, the MAX IV data catalog will permanently store the experimental metadata and provide a persistent identifier for each data collection.

Data will be replicated, using a dedicated network, onto a separate storage system at the Lund University computing center (Lunarc). The replicated data at the Lunarc site can be efficiently transferred to offline resources at Lunarc or to other SNIC sites using the 100-GB/s SUNET C network within the 3-month retention period. This setup also ensures that the user data transfer does not impact the experiment at the beamlines.

For a scientist, it is crucial how measured data can be reached. Besides having a basic access to data in the beamline, “expert” users will be able to handle data by algorithms and computational methods at the computing cluster during the experiment. With longer latencies, scientists can run analysis at Lunarc and later after the experiment from SNIC. Providing user access to computing resources is a mission and know-how of these big infrastructures.

Our initial analysis environment will serve to the first operating beamlines (one for protein crystallography and NanoMAX for imaging and microscopy). The underlying, scalable, infrastructure is using the high-performance IBM Spectrum Scale file system and is connected to a computing cluster with tailored resources for each experiment. A typical setup on the compute cluster for an imaging beamline is 200 cores, 4 GPU nodes and 400 TB storage. The system will be accessible via terminal or a remote desktop solution using full hardware acceleration.

### Timing the acquisition

An obvious asset of imaging in full-field mode (and the near-field diffraction regime) is the simple and robust experimental setup, flexible sample environments but most importantly a uniquely fast acquisition of a large 3D dataset [16]. The timing of such experiments is critical and is up to now only weakly correlated with the dynamics in the sample. One reason is that there is practically no feedback available at these acquisition rates. Our concept of real-time data monitoring at the imaging beamline at MAX IV will enable an informed decision taking process in terms of acquisition control and raw data recording. This step is critical for ensuring that only relevant raw data are saved and enter the offline analysis pipeline. One way to achieve this is through fast tomographic reconstruction and feature separation (image binarization) with consequent basic topological and statistical measurement on selected tomographic slices.

## Image analysis

### Tomographic and phase reconstruction platform

At synchrotron facilities, the image reconstruction (tomographic and phase) is often provided using one single method that has proven to be robust. There are no enough resources or know-how directly at the facilities to develop or simply implement existing multiple advanced reconstruction methods. We think that a platform based on the recently developed Operation Discretisation Library (ODL) [17] in python will stimulate the development and implementation of modern tomographic and phase reconstruction schemes to reflect the variable experimental settings. In particular, this approach will favor the construction of iterative schemes combining tomography and phase retrieval in the near-field (tomographic microscopy) and far-field (ptychography) approximation. Currently, the library includes the Astra toolbox and the Gridrec forward projector [18]. When completed by most of the modern and also traditional tomographic and phase reconstruction operators and when the support of large data volumes and compatibility with real-time data monitoring is enabled, the ODL will be a valuable tool at the imaging beamlines for fast prototyping to facilitate advances.

Currently, there are three distinct groups of tomographic reconstruction routines implemented at the Lunarc GPU cluster. One being a Log-polar fast Radon transform approach developed at the Mathematics Department at Lund University [20], the second being algebraic methods such as SIRT and the third group being discrete methods such as DART and an energy minimization method [21]. The latter are more computationally intensive and therefore slower. But useful for specific cases in particular when a small number of higher quality projections are available. Contrary, the first method is fast and qualifies for on-the-fly data analysis during experiment making it particularly valuable for processing time resolved acquisition. Performance of all the methods in terms of speed is evaluated in Table 1, taken from [20].

**Table 1:** Benchmarking the fast radon transform algorithm implemented at the Lunarc GPU cluster

Method	1024	2048
LP/GPU	1.2 10^−2^	4.5 10^−2^
FBP (ASTRA)/GPU	4.3 10^−2^	2.8 10^−1^
LP/CPU	1.1 10^−1^	4 10^−1^
IRT/CPU	1.7	1.2 10^1^

The data are taken from [20] where the authors compared among others the following four methods:* LP* Log-Polar Radon transform and backprojection developed by the authors, FBP as implemented in the Astra toolbox [19],* IRT* Image Reconstruction Toolbox [22]

Each algorithm is evaluated for the image size of 1024 and 2048 pixels (values represent computational time in seconds)

**Fig. 2:**
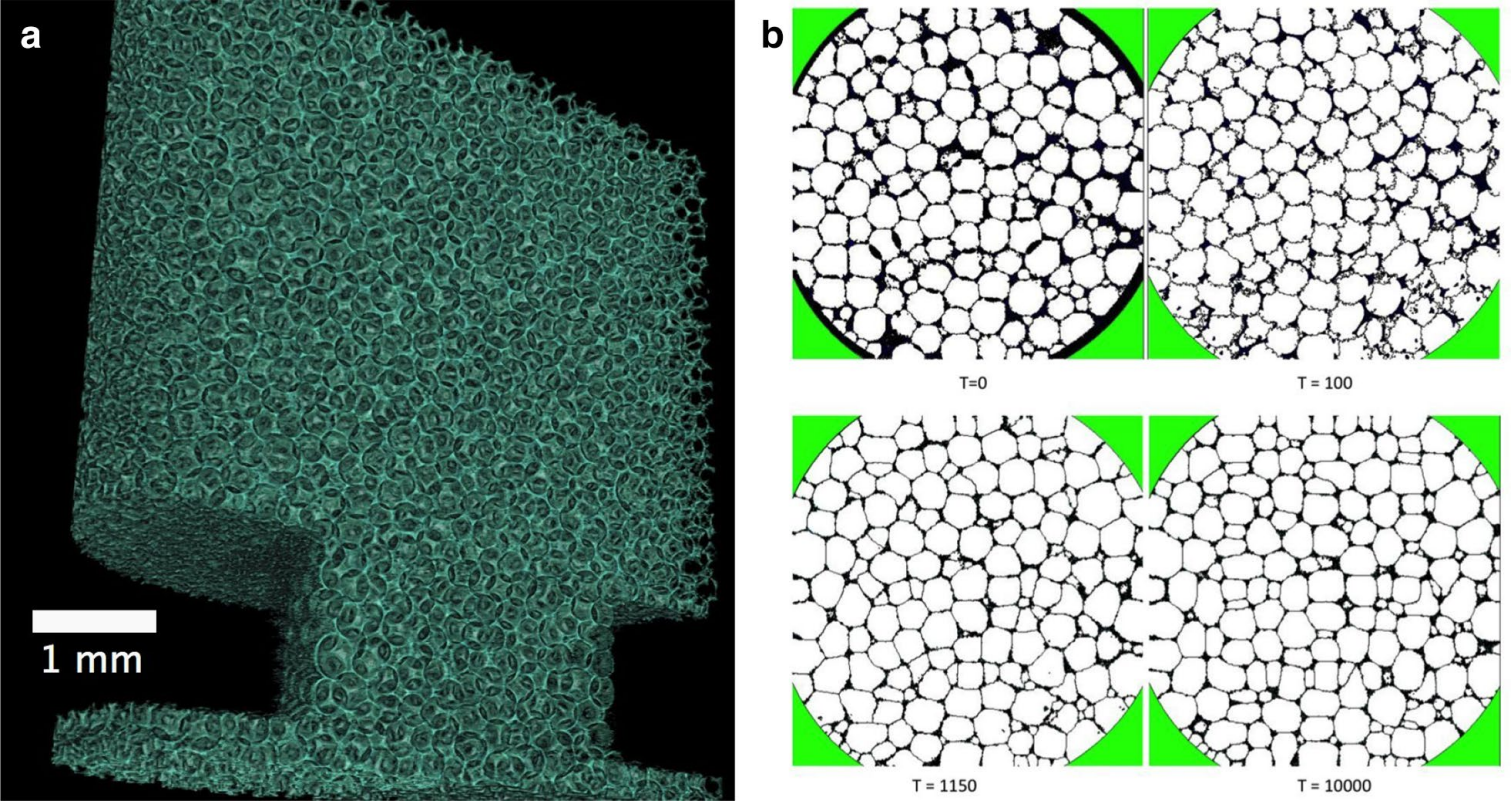
The refinement of the cell structure analysis performed on a liquid foam [26]. **a** The 3D volume rendering of the liquid phase is presented. **b** The four stages of refinement. The four cross sections in **b** show the results of CompuCell3D simulations at Monte Carlo steps: 0, 100, 1150 and 10000. The initial structure (*T=0*) is derived from the experimental tomographic slice and the simulation is stopped at Monte Carlo step 10000

### Quantitative analysis

The acquisition of time resolved three-dimensional datasets brings several major challenges. First, the more precise in time and space we are becoming the larger becomes the data size, surpassing easily several TB when following one single dynamic process lasting less then 10 min. Second, new user communities are attracted to synchrotron facilities by the unique capabilities coming up, offered by the higher brightness and faster detectors. These users have typically very limited or non-existent help for data analysis. Some guidance is traditionally offered in the spare time of the facility staff, but in the majority of cases the user is left alone with the task to find ways to access and retrieve the relevant information. Third, it is imperative that quantitative results emerge for these facilities to become a routine tool of use for materials design and structural biology, among others.

One way of dealing with the high demand for feature extraction tools from multi-dimensional images is to introduce showcases comprising typical features re-occurring in various scientific fields. One example is cellular systems. A lot of effort has been put into the development of quantitative analysis tools of complex cellular systems in the past [23], yet no reliable single tool exists today that would give a confidence level better than a few percents in the quantification of the topology and texture of this type of images. In the case of liquid foams, the main reason is that the resolution of dynamic tomographic microscopy is insufficient to resolve the films between individual cells, yet the analysis imperatively expects each single cell uniquely labeled to distinguish the boundaries between them. The new direction which we think has a potential to help in this is to refine the outcome of basic image processing by modeling the system using Monte Carlo methods. We used Cellular Potts Model [24], in the framework of the open source software CompuCell3D [25] one can reconstruct the correct foam from the tomographic images and to obtain a realistic description of the foam topology in 3D. Foam behavior and structure are determined by physical boundaries; in particular, the surface tension is the main contribution to the total entropy of a foam structure. Starting from a rough initial guess, the individual cell wall positions predicted by simple image processing in a liquid foam are refined using the Cellular Potts Model in Fig. 2 by minimizing the surface tension. Bringing physical laws into the image processing workflow is an attractive opportunity that is likely to help in gaining confidence in the quantification platforms applied to complex multi-dimensional data. For a number of selected shapes, basic but robust quantitative image analysis workflows should be developed to guide the users of imaging facilities toward an efficient and reliable quantification of acquired images.

## Conclusions

The high coherent flux of the MAX IV laboratory will be best used to advance time resolved experiments to reach a performance never seen before. The fast data acquisition will be inevitably accompanied by high data flow rates which will be sustainable only if adequate data reduction schemes are going to be established. The data handling concept at MAX IV has been designed such that it will support real-time data visualization and promote remote offline data analysis through the off-site computing clusters. The mirroring of data analysis packages to these clusters will enable simplified access to the information content of the multidimensional images.

## References

[1] Eriksson MJ, van der Veen F, Quitmann C (2014). Diffraction-limited storage rings a window to the science of tomorrow. J. Synchrotron. Radiat..

[2] The HDF Group, Hierarchical Data Format, version 5, 1997–2016. https://www.hdfgroup.org. Accessed 24 Oct 2016

[3] Könnecke M, Akeroyd FA, Bernstein HJ, Brewster AS, Campbell SI, Clausen B, Cottrell S, Hoffmann JU, Jemian PR, Mannicke D, Osborn R, Peterson PF, Richter T, Suzuki J, Watts B, Wintersberger E, Wuttke J (2015). The NeXus data format. J. Appl. Cryst..

[4] De Carlo F, Gürsoy D, Marone F, Rivers M, Parkinson DY, Khan F, Schwarz N, Vine DJ, Vogt S, Gleber SC, Narayanan S, Newville M, Lanzirotti T, Sun Y, Hong YP, Jacobsen C (2014). Scientific data exchange: a schema for HDF5-based storage of raw and analyzed data. J. Synchrotron. Radiat..

[5] Maia FR (2012). The coherent X-ray imaging data bank. Nat. Methods..

[6] Mokso, R., Schleputz, C., Billich, H., Theidel, G., Marone, F., Celcer, T., Mikuljan, G., Schmidt, E., Schlumpf, N., Stampanoni, M.: GigaFRoST: Gigabit Fast Readout System for Tomography. J. Synchrotron Radiat. (2016) (in preparation) 10.1107/S1600577517013522 PMC5665295 29091068

[7] Damiani D, Dubrovin M, Gaponenko I, Kroeger W, Lane TJ, Mitra A, O’Grady CP, Salnikov A, Sanchez-Gonzalez A, Schneider D, Yoon CH (2016). Linac coherent light source data analysis using psana. J. Appl. Crystallogr..

[8] Sjöstrom A, Lindemann J, Church R (2011). The experience of GPU calculations at Lunarc. Proc. SPIE..

[9] Johnsson, L., Ahlin, D., Wang, J.: The SNIC/KTH PRACE prototype: achieving high energy efficiency with commodity technology without acceleration. In: Green computing conference, IEEE, 87–95 (2010)

[10] Coutinho, T., Cuní, G., Fernández-Carreiras, D., Klora, J., Pascual-Izarra, C., Reszela, Z., Suñé, R., Homs, A., Taurel, E., Rey, V.: SARDANA: the software for building SCADAS in scientific environments. In: Proceedings of ICALEPCS2011, Grenoble, WEAAUST01, 607–609 (2011)

[11] Taurel, E., Fernandez, D., Ounsy, M., Scafuri, C.: The TANGO Collaboration Status and Some of The Latest Developments. In: Proceedings of the 10th ICALEPCS conference, ICALEPCS2005, WE2.3-6O, 1–6 (2005)

[12] Taurus python framework for control and data acquisition: https://www.taurus-scada.org Accessed 14 Aug 2016

[13] Petitdemange, S., Claustre, L., Homs-Puron, A., Papillon, E., Homs-Regojo, R.: The LIMA project update. In: Proceedings of ICALEPCS2013, San Francisco, FRCOAAB08, 1489–1492 (2013)

[14] Marchesini S, Krishnan H, Daurer BJ, Shapiro DA, Perciano T, Sethian JA, Maia FR (2016). SHARP: a distributed GPU-based ptychographic solver. J. Appl. Crystallogr..

[15] High data-rate macromolecular crystallography project https://hdrmx.medsbio.org Accessed 14 Aug 2016

[16] Mokso R, Marone F, Irvine S, Nyvlt M, Schwyn D, Mader K, Taylor G, Krapp H, Skeren M, Stampanoni M (2013). Advantages of phase retrieval in fast tomographic microscopy. J. Phys. D.

[17] Operation Discretisation Library, KTH Stockholm https://www.github.com/odlgroup/odl Accessed 2 Sept 2016

[18] Arcadu, F., Nilchian, M., Studer, A., Stampanoni, M., Marone, F.: A forward regridding method with minimal oversampling for accurate and efficient iterative tomographic algorithms. IEEE. Trans. Image. Process. (2016) (in preparation). 10.1109/TIP.2016.2516945 26800537

[19] Palenstijn WJ, Batenburg KJ, Sijbers J (2011). Performance improvements for iterative electron tomography reconstruction using graphics processing units (GPUs). J. Struct. Biol..

[20] Andersson F, Carlsson M, Nikitin V (2016). Fast algorithms and efficient GPU implementations for the Radon transform and the back-projection operator represented as convolution operators. SIAM. J. Imaging Sci..

[21] Varga L, Balazs P, Nagy A (2015). Discrete tomographic reconstruction via adaptive weighting of gradient descents. Comput. Methods. Biomech. Biomed. Eng..

[22] Fessler, J.: Image reconstruction toolbox. University of Michigan, Ann Arbor, Michigan, USA. http://web.eecs.umich.edu/fessler/irt/fessler.tgz Accessed 2 Sept 2016

[23] Mader K, Mokso R, Raufaste C, Dollet B, Santucci S, Lambert J, Stampanoni M (2012). Quantitative 3D characterization of cellular materials: segmentation and morphology of foam. Colloids. Surf. A..

[24] Graner F, Glazier JA (1992). Simulation of biological cell sorting using a two-dimensional extended Potts model. Phys. Rev. Lett..

[25] Swat M, Thomas GL, Belmonte JM, Shirinifard A, Hmeljak D, Glazier JA (2012). Multi-scale modeling of tissues using CompuCell 3D. Methods. Cell. Biol..

[26] Raufaste C, Dollet B, Santucci S, Mader K, Mokso R (2015). Three-dimensional foam flow resolved by fast X-ray tomographic microscopy. Euro. Phys. Lett..

